# Supplementary material for: Antibodies response in symptomatic and asymptomatic SARS-CoV-2 infected persons in Thailand
Source: PLoS One. 2025 Feb 11;20(2):e0308850. doi: 10.1371/journal.pone.0308850 (PMC11813072; doi:10.1371/journal.pone.0308850)
Supplement: S1 Protocol — (DOCX) [file pone.0308850.s001.docx]

### S1 Protocol. This is the S1 Protocol ELISA of sera to detect for antibodies against 2019-nCoV protein (IgG)

### Materials

96 well NUNC maxisorp plates (NUNC, Cat no: 442404)

Adhesive sheet (for covering plate)

Coating antigen

Sera for testing (aliquot in BSC, heat-inactivated for 56^o^C for 30 minutes)

Anti-human IgG-HRP (Santa Cruz, Cat no: SC-2907 *this may not be available anymore by the company)

TMB ELISA substrate (Life technologies, Cat no: 002023)

Stop solution (KPL sera care, Cat no: 5150-0019)

PBS+0.05% Tween20 (PBST)

Blocking buffer (BD Pharmingen, BD OptEIA assay diluent, Cat no: 51-2641KC; we buy from Zuellig pharma, Cat no: 555213)

Coating buffer (Bicarbonate buffer: 0.015M Na_2_CO_3_, 0.035M NaHCO_3_, pH 9.6)

### Methods

1. Coat the 96 well plate with 2ug/ml of 2019-nCoV protein diluted in bicarbonate buffer at 50ul/well in 4^o^C overnight.
2. The next day, remove the coating solution as biohazard waste. Wash the plate 5x by filling each well with 150ul of PBST and remove the PBST wash as biohazard waste.
3. Remove remaining solutions by tapping it hard against a paper towel.
4. Block the remaining protein binding sites with 150ul blocking buffer per well. Incubate the plate at room temperature for 2 hours.
5. Wash the plate 1x with PBST wash buffer and remove excess solutions as steps 2-3.
6. Add heat-inactivated serum diluted 1:100 in blocking buffer in duplicates, at 50ul per well. Incubate the plate at room temperature for 2 hours.
7. Wash plate 5x with PBST wash buffer and remove excess solutions as steps 2-3.
8. Add anti-human IgG-HRP diluted 1:2000 in blocking buffer, at 50ul per well. Incubate the plate at room temperature for 1 hour.
9. Wash plate 5x with PBST wash buffer and remove excess solutions as step 2-3.
10. Add 50ul of TMB substrate per well. Observe the chromogenic reaction and stop after 1 minute before it reaches the maximum readable range of the Cytation5 plate reader (i.e. OD450 of 2).
11. Stop reaction by addition of 50ul per well of Stop solution.
12. Read the absorbance on the Cytation5 plate reader. TMB – 450um, background of plate – 570um.
