# Supplementary material for: Antibodies response in symptomatic and asymptomatic SARS-CoV-2 infected persons in Thailand
Source: PLoS One. 2025 Feb 11;20(2):e0308850. doi: 10.1371/journal.pone.0308850 (PMC11813072; doi:10.1371/journal.pone.0308850)
Supplement: S2 Protocol — (DOCX) [file pone.0308850.s002.docx]

**S2 Protocol. This is the S2** **Protocol Capture ELISA to detect for IgM antibodies against 2019-nCoV protein**

### Materials

96 well NUNC maxisorp plates (NUNC, Cat no: 442404)

Adhesive sheet (for covering plate)

Coating antigen

Sera for testing (aliquot in BSC, heat-inactivated for 56^o^C for 30 minutes)

Goat Anti-human IgM (SeraCare, Cat no: 5210-0157, stock concentration = 1mg/ml)

TMB ELISA substrate (Life technologies, Cat no: 002023)

Stop solution (KPL sera care, Cat no: 5150-0019)

PBS+0.05% Tween20 (PBST)

Blocking buffer (BD Pharmingen, BD OptEIA assay diluent, Cat no: 51-2641KC; we buy from Zuellig pharma, Cat no: 555213)

Coating buffer (Bicarbonate buffer: 0.015M Na_2_CO_3_, 0.035M NaHCO_3_, pH 9.6)

Negative controls (naïve human serum)

RBD-HRP (Genscript, stock concentration 0.38mg/ml)

### Methods

1. Coat the 96 well plate with 10ug/ml of goat anti-human IgM antibody diluted in bicarbonate buffer (for 1 full plate, add 50ul of antibody to 5ml of coating buffer) at 50ul/well in 4^o^C overnight.
2. The next day, remove the coating solution as biohazard waste. Wash the plate 5x by filling each well with 150ul of PBST and remove the PBST wash as biohazard waste.
3. Remove remaining solutions by tapping it hard against a paper towel.
4. Block the remaining protein binding sites with 150ul blocking buffer per well. Incubate the plate at room temperature for 2 hours.
5. Wash the plate 1x with PBST wash buffer and remove excess solutions as steps 2-3.
6. Add heat-inactivated serum diluted 1:50 in blocking buffer in duplicates, at 50ul per well. Include negative controls. Incubate the plate at room temperature for 2 hours or at 37 deg for 1 hour.
7. Wash plate 5x with PBST wash buffer and remove excess solutions as steps 2-3.
8. Add RBD-HRP diluted to 4ug/ml in blocking buffer (stock concentration = 0.38mg/ml, for 1 full plate, add 52ul to 5ml of blocking buffer), at 50ul per well. Incubate the plate at room temperature for 1 hour or 37 deg for half an hour.
9. Wash plate 5x with PBST wash buffer and remove excess solutions as step 2-3.
10. Add 50ul of TMB substrate per well. Observe the chromogenic reaction and stop after 2 minutes before it reaches the maximum readable range of the Cytation5 plate reader (i.e. OD450 of 2).
11. Stop reaction by addition of 50ul per well of Stop solution.
12. Read the absorbance on the Cytation5 plate reader immediately after stopping plate. TMB – 450um, background of plate – 570um. *Note: if there are multiple plates, you should work on one plate at a time. Leave the last wash buffer in the other plates and only proceed with adding TMB solution one plate at a time.
