# Supplementary figures and images for: Antibodies response in symptomatic and asymptomatic SARS-CoV-2 infected persons in Thailand
Source: PLoS One. 2025 Feb 11;20(2):e0308850. doi: 10.1371/journal.pone.0308850 (PMC11813072; doi:10.1371/journal.pone.0308850)

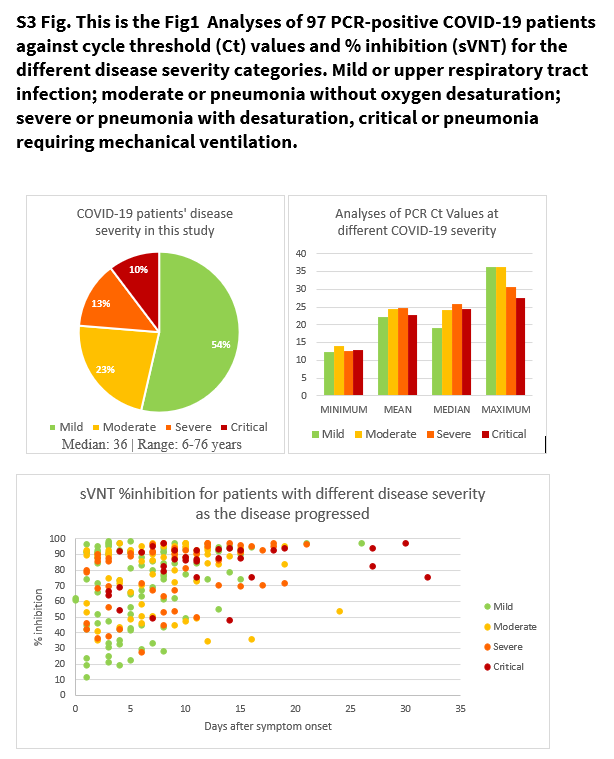

Supplement: S1 Fig — Mild or upper respiratory tract infection; moderate or pneumonia without oxygen desaturation; severe or pneumonia with desaturation, critical or pneumonia requiring mechanical ventilation. (TIF) [file pone.0308850.s003.tif]

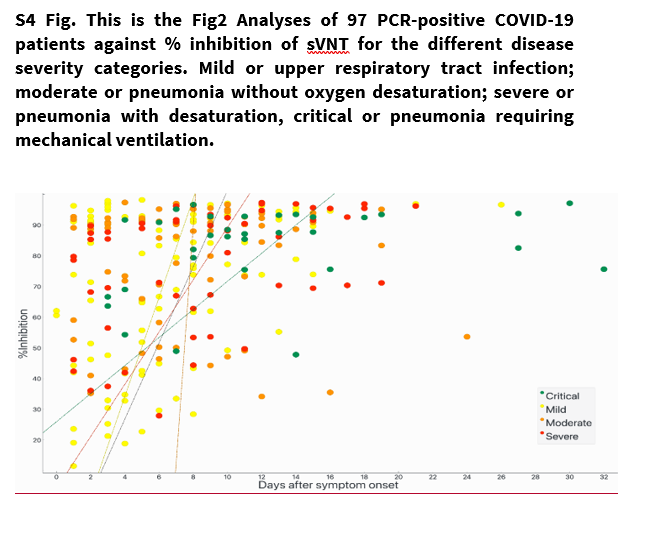

Supplement: S2 Fig — Mild or upper respiratory tract infection; moderate or pneumonia without oxygen desaturation; severe or pneumonia with desaturation, critical or pneumonia requiring mechanical ventilation. (TIF) [file pone.0308850.s004.tif]

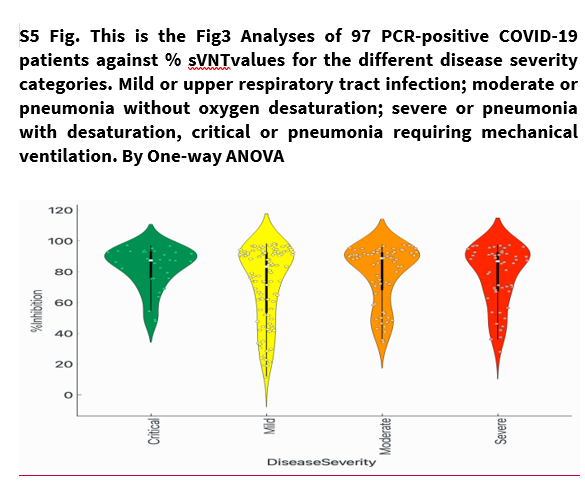

Supplement: S3 Fig — Mild or upper respiratory tract infection; moderate or pneumonia without oxygen desaturation; severe or pneumonia with desaturation, critical or pneumonia requiring mechanical ventilation by One-way ANOVA. (TIF) [file pone.0308850.s005.tif]
