# Supplementary material for: Antibodies response in symptomatic and asymptomatic SARS-CoV-2 infected persons in Thailand
Source: PLoS One. 2025 Feb 11;20(2):e0308850. doi: 10.1371/journal.pone.0308850 (PMC11813072; doi:10.1371/journal.pone.0308850)
Supplement: S1 Table — (DOCX) [file pone.0308850.s006.docx]

S6 Table. This is the table1 shows The results of samples from PCR-positive COVID-19 patients in the first half of 2020 and shows the results of samples from State Quarantine, from persons travelling back from Sudan in the latter half of 2020 tested using rRT-PCR, ELISA IgG, IgM and sVNT.

| Patient ID | Sex | Age | Ct value from rRT-PCR | Disease Severity | Specimen collection date | Days after symptom onset | Days after exposure | Assay | | | |
| --- | --- | --- | --- | --- | --- | --- | --- | --- | --- | --- | --- |
|  |  |  |  |  |  |  |  | Neutralization Ab (sVNT) | % Inhibition | IgM | IgG |
| PT001 | M | 76 | 12.89 | Critical | 2020-03-20 | 11 | N/A | Positive | 85.40 | Positive | Positive |
|  |  |  |  |  | 2020-03-22 | 13 | N/A | Positive | 87.52 | Positive | Positive |
|  |  |  |  |  | 2020-03-24 | 15 | N/A | Positive | 87.73 | Positive | Positive |
|  |  |  |  |  | 2020-03-28 | 19 | N/A | Positive | 93.45 | Positive | Positive |
|  |  |  |  |  | 2020-04-05 | 27 | N/A | Positive | 93.77 | Positive | Positive |
| PT002 | M | N/A | 26.39 | Critical | 2020-03-16 | 6 | N/A | Positive | 90.90 | Positive | Positive |
|  |  |  |  |  | 2020-03-21 | 11 | N/A | Positive | 92.85 | Negative | Positive |
| PT003 | M | N/A | 14.06 | Mild | 2020-03-22 | 2 | N/A | Positive | 87.87 | Positive | Positive |
| PT004 | M | N/A | 14.61 | Mild | 2020-03-13 | 2 | N/A | Positive | 84.16 | Positive | Positive |
|  |  |  |  |  | 2020-03-20 | 9 | N/A | Positive | 84.16 | Positive | Positive |
|  |  |  |  |  | 2020-03-22 | 11 | N/A | Positive | 84.37 | Positive | Positive |
| PT005 | F | N/A | 34.88 | Mild | 2020-03-14 | 3 | N/A | Positive | 95.87 | Positive | Positive |
| PT006 | M | N/A | 16.47 | Mild | 2020-03-15 | 3 | N/A | Positive | 94.88 | Positive | Positive |
|  |  |  |  |  | 2020-03-22 | 10 | N/A | Positive | 93.31 | Positive | Positive |
|  |  |  |  |  | 2020-03-21 | 9 | N/A | Positive | 93.41 | Positive | Positive |
| PT007 | F | N/A | 26.75 | Mild | 2020-03-16 | 14 | N/A | Positive | 95.62 | Positive | Positive |
| PT008 | F | N/A | 19.74 | Mild | 2020-03-16 | 9 | N/A | Positive | 96.61 | Positive | Positive |
| PT009 | M | N/A | 19.68 | Moderate | 2020-03-16 | 2 | 4 | Positive | 35.22 | Positive | Positive |
|  |  |  |  |  | 2020-03-24 | 10 | 12 | Positive | 47.12 | Positive | Positive |
|  |  |  |  |  | 2020-03-26 | 12 | 14 | Positive | 34.16 | Positive | Positive |
|  |  |  |  |  | 2020-03-30 | 16 | 18 | Positive | 35.50 | Positive | Positive |
| PT010 | M | 50 | 26.76 | Critical | 2020-03-16 | 3 | N/A | Positive | 63.63 | Positive | Positive |
|  |  |  |  |  | 2020-03-22 | 9 | N/A | Positive | 86.65 | Positive | Positive |
| PT011 | M | 32 | 22.32 | Moderate | 2020-03-16 | 9 | N/A | Positive | 72.17 | Positive | Positive |
| PT012 | F | 18 | 23.07 | Mild | 2020-03-18 | 6 | N/A | Positive | 29.65 | Positive | Positive |
|  |  |  |  |  | 2020-03-20 | 8 | N/A | Positive | 28.41 | Positive | Positive |
|  |  |  |  |  | 2020-03-21 | 9 | N/A | Positive | 61.94 | Positive | Positive |
| PT013 | F | 24 | 25.42 | Severe | 2020-03-18 | 9 | N/A | Positive | 92.73 | Positive | Positive |
|  |  |  |  |  | 2020-03-21 | 12 | N/A | Positive | 97.33 | Positive | Positive |
|  |  |  |  |  | 2020-03-25 | 16 | N/A | Positive | 95.42 | Positive | Positive |
|  |  |  |  |  | 2020-03-27 | 18 | N/A | Positive | 96.89 | Positive | Positive |
| PT014 | F | 35 | 15.97 | Mild | 2020-03-18 | 4 | N/A | Positive | 41.66 | Positive | Positive |
|  |  |  |  |  | 2020-03-21 | 7 | N/A | Positive | 68.93 | Positive | Positive |
| PT015 | F | 47 | 23.17 | Mild | 2020-03-18 | 4 | N/A | Positive | 32.76 | Positive | Positive |
|  |  |  |  |  | 2020-03-21 | 7 | N/A | Positive | 33.49 | Positive | Positive |
|  |  |  |  |  | 2020-03-22 | 8 | N/A | Positive | 43.36 | Positive | Positive |
|  |  |  |  |  | 2020-03-24 | 10 | N/A | Positive | 49.21 | Positive | Positive |
| PT016 | M | 25 | N/A | Mild | 2020-03-18 | 2 | N/A | Positive | 65.46 | Positive | Positive |
|  |  |  |  |  | 2020-03-21 | 5 | N/A | Positive | 64.75 | Positive | Positive |
| PT017 | M | 37 | 36.26 | Mild | 2020-03-18 | 3 | N/A | Positive | 21.30 | Positive | Positive |
|  |  |  |  |  | 2020-03-20 | 5 | N/A | Positive | 22.73 | Positive | Positive |
|  |  |  |  |  | 2020-03-21 | 6 | N/A | Positive | 44.89 | Positive | Positive |
| PT018 | M | 30 | 29.83 | Mild | 2020-03-19 | 8 | 12 | Positive | 73.80 | Positive | Positive |
|  |  |  |  |  | 2020-03-22 | 11 | 15 | Positive | 73.66 | Positive | Positive |
|  |  |  |  |  | 2020-03-23 | 12 | 16 | Positive | 73.82 | Positive | Positive |
|  |  |  |  |  | 2020-03-26 | 15 | 19 | Positive | 73.93 | Positive | Positive |
| PT019 | F | 48 | 27.91 | Severe | 2020-03-22 | 2 | 11 | Positive | 68.18 | Positive | Positive |
|  |  |  |  |  | 2020-03-23 | 3 | 12 | Positive | 69.56 | Positive | Positive |
|  |  |  |  |  | 2020-03-27 | 7 | 16 | Positive | 66.97 | Positive | Positive |
|  |  |  |  |  | 2020-03-29 | 9 | 18 | Positive | 67.26 | Positive | Positive |
|  |  |  |  |  | 2020-04-02 | 13 | 22 | Positive | 70.33 | Positive | Positive |
|  |  |  |  |  | 2020-04-04 | 15 | 24 | Positive | 69.44 | Positive | Positive |
|  |  |  |  |  | 2020-04-06 | 17 | 26 | Positive | 70.37 | Positive | Positive |
|  |  |  |  |  | 2020-04-08 | 19 | 28 | Positive | 71.17 | Positive | Positive |
| PT020 | F | 23 | 17.1 | Mild | 2020-03-20 | 2 | N/A | Positive | 87.79 | Positive | Positive |
|  |  |  |  |  | 2020-03-22 | 4 | N/A | Positive | 73.44 | Positive | Positive |
|  |  |  |  |  | 2020-03-24 | 6 | N/A | Positive | 62.72 | Positive | Positive |
| PT021 | M | 32 | 15.7 | Mild | 2020-03-20 | 5 | N/A | Positive | 91.38 | Positive | Positive |
|  |  |  |  |  | 2020-03-23 | 8 | N/A | Positive | 91.83 | Positive | Positive |
| PT022 | F | 52 | 31.68 | Moderate | 2020-03-20 | 1 | N/A | Positive | 91.88 | Positive | Positive |
|  |  |  |  |  | 2020-03-22 | 3 | N/A | Positive | 92.29 | Positive | Positive |
|  |  |  |  |  | 2020-03-24 | 5 | N/A | Positive | 92.28 | Positive | Positive |
|  |  |  |  |  | 2020-03-28 | 9 | N/A | Positive | 92.65 | Positive | Positive |
|  |  |  |  |  | 2020-03-30 | 11 | N/A | Positive | 92.70 | Positive | Positive |
|  |  |  |  |  | 2020-03-31 | 12 | N/A | Positive | 93.77 | Positive | Positive |
| PT023 | M | 39 | 18.87 | Moderate | 2020-03-21 | 1 | 7 | Positive | 89.12 | Positive | Positive |
|  |  |  |  |  | 2020-03-22 | 2 | 8 | Positive | 90.14 | Positive | Positive |
|  |  |  |  |  | 2020-03-23 | 3 | 9 | Positive | 90.35 | Positive | Positive |
|  |  |  |  |  | 2020-03-27 | 7 | 13 | Positive | 90.24 | Positive | Positive |
|  |  |  |  |  | 2020-03-29 | 9 | 15 | Positive | 90.75 | Positive | Positive |
| PT024 | F | 21 | 18.67 | Mild | 2020-03-21 | 5 | N/A | Positive | 42.55 | Positive | Positive |
| PT025 | M | 27 | 15.17 | Mild | 2020-03-21 | 3 |  | Positive | 47.58 | Positive | Positive |
| PT026 | M | 51 | 27.98 | Moderate | 2020-03-21 | 9 | 15 | Positive | 95.52 | Positive | Positive |
|  |  |  |  |  | 2020-03-25 | 13 | 19 | Positive | 83.43 | Positive | Positive |
|  |  |  |  |  | 2020-03-31 | 19 | 25 | Positive | 83.35 | Positive | Positive |
| PT027 | M | 27 | 14.06 | Mild | 2020-03-24 | 2 | N/A | Positive | 91.07 | Positive | Positive |
| PT028 | M | 59 | 21.11 | Severe | 2020-03-22 | 2 | N/A | Positive | 85.32 | Positive | Positive |
|  |  |  |  |  | 2020-03-26 | 6 | N/A | Positive | 71.28 | Positive | Positive |
|  |  |  |  |  | 2020-03-30 | 10 | N/A | Positive | 88.20 | Positive | Positive |
|  |  |  |  |  | 2020-04-01 | 12 | N/A | Positive | 96.96 | Positive | Positive |
| PT029 | F | 33 | 27.35 | Mild | 2020-03-23 | 7 | 8 | Positive | 79.43 | Positive | Positive |
|  |  |  |  |  | 2020-03-24 | 8 | 9 | Positive | 84.39 | Positive | Positive |
|  |  |  |  |  | 2020-03-26 | 10 | 12 | Positive | 97.07 | Positive | Positive |
| PT030 | M | 28 | 16.56 | Mild | 2020-03-23 | 4 | N/A | Positive | 97.35 | Positive | Positive |
| PT031 | M | 38 | 18.73 | Mild | 2020-03-24 | 2 | N/A | Positive | 94.77 | Positive | Positive |
| PT032 | F | 23 | 33.2 | Moderate | 2020-03-24 | 4 | N/A | Positive | 97.39 | Positive | Positive |
|  |  |  |  |  | 2020-03-30 | 10 | N/A | Positive | 96.70 | Positive | Positive |
| PT033 | F | 36 | 33.42 | Mild | 2020-03-24 | 2 | N/A | Positive | 91.50 | Positive | Positive |
| PT034 | F | 52 | 19.53 | Moderate | 2020-03-24 | 4 | N/A | Positive | 71.84 | Positive | Positive |
|  |  |  |  |  | 2020-03-26 | 6 | N/A | Positive | 95.21 | Positive | Positive |
|  |  |  |  |  | 2020-03-30 | 10 | N/A | Positive | 94.41 | Positive | Positive |
|  |  |  |  |  | 2020-04-01 | 12 | N/A | Positive | 94.34 | Positive | Positive |
| PT035 | F | 25 | 19.8 | Moderate | 2020-03-24 | 2 | N/A | Positive | 88.78 | Positive | Positive |
|  |  |  |  |  | 2020-03-26 | 4 | N/A | Positive | 73.37 | Positive | Positive |
|  |  |  |  |  | 2020-03-29 | 7 | N/A | Positive | 97.01 | Positive | Positive |
|  |  |  |  |  | 2020-04-01 | 10 | N/A | Positive | 96.64 | Positive | Positive |
| PT036 | F | 38 | 34.88 | Mild | 2020-03-25 | 3 | N/A | Positive | 97.92 | Positive | Positive |
|  |  |  |  |  | 2020-03-27 | 5 | N/A | Positive | 91.63 | Positive | Positive |
| PT037 | F | 35 | 18.25 | Mild | 2020-03-26 | 5 | N/A | Positive | 98.20 | Positive | Positive |
| PT038 | F | 39 | 18.3 | Moderate | 2020-03-26 | 3 | N/A | Positive | 74.76 | Positive | Positive |
|  |  |  |  |  | 2020-03-28 | 5 | N/A | Positive | 91.72 | Positive | Positive |
| PT039 | M | 40 | 20.61 | Mild | 2020-03-26 | 3 | N/A | Positive | 97.11 | Positive | Positive |
| PT040 | F | 20 | 35.19 | Mild | 2020-03-27 | 3 | N/A | Positive | 96.83 | Positive | Positive |
| PT041 | M | 35 | 34.89 | Mild | 2020-03-27 | 3 | N/A | Positive | 90.72 | Positive | Positive |
| PT042 | F | 21 | 36.22 | Mild | 2020-03-27 | 1 | N/A | Negative | 19.12 | Positive | Positive |
|  |  |  |  |  | 2020-03-29 | 3 | N/A | Positive | 25.25 | Positive | Positive |
| PT043 | M | 32 | 35.91 | Mild | 2020-03-27 | 4 | N/A | Negative | 18.85 | Positive | Positive |
| PT044 | F | 23 | 15.12 | Mild | 2020-03-28 | 5 | N/A | Positive | 51.84 | Positive | Positive |
| PT045 | F | 36 | 36.29 | Moderate | 2020-03-29 | 6 | N/A | Positive | 50.26 | Positive | Positive |
|  |  |  |  |  | 2020-03-30 | 7 | N/A | Positive | 50.08 | Positive | Positive |
|  |  |  |  |  | 2020-04-16 | 24 | N/A | Positive | 53.63 | Positive | Positive |
| PT046 | F | 36 | 13.97 | Moderate | 2020-03-29 | 6 | N/A | Positive | 58.27 | Positive | Positive |
|  |  |  |  |  | 2020-04-01 | 9 | N/A | Positive | 44.28 | Positive | Positive |
| PT047 | M | 36 | 21.49 | Mild | 2020-03-30 | 4 | N/A | Positive | 92.70 | Positive | Positive |
| PT048 | F | 41 | 19.43 | Moderate | 2020-03-31 | 1 | N/A | Positive | 52.66 | Positive | Positive |
|  |  |  |  |  | 2020-04-02 | 3 | N/A | Positive | 89.06 | Positive | Positive |
| PT049 | M | 36 | 22.66 | Critical | 2020-03-31 | 4 | N/A | Positive | 54.30 | Positive | Positive |
|  |  |  |  |  | 2020-04-03 | 7 | N/A | Positive | 48.93 | Positive | Positive |
|  |  |  |  |  | 2020-04-07 | 11 | N/A | Positive | 75.47 | Positive | Positive |
|  |  |  |  |  | 2020-04-12 | 16 | N/A | Positive | 75.59 | Positive | Positive |
|  |  |  |  |  | 2020-04-28 | 32 | N/A | Positive | 75.63 | Positive | Positive |
| PT050 | M | 54 | 12.63 | Severe | 2020-04-01 | 2 | N/A | Positive | 36.08 | Positive | Positive |
|  |  |  |  |  | 2020-04-05 | 6 | N/A | Positive | 27.91 | Positive | Positive |
|  |  |  |  |  | 2020-04-07 | 8 | N/A | Positive | 44.37 | Positive | Positive |
|  |  |  |  |  | 2020-04-10 | 11 | N/A | Positive | 49.69 | Positive | Positive |
| PT051 | M | 21 | 27.69 | Mild | 2020-04-01 | 3 | N/A | Positive | 30.42 | Positive | Positive |
|  |  |  |  |  | 2020-04-04 | 6 | N/A | Positive | 88.33 | Positive | Positive |
| PT052 | F | 31 | 34.63 | Mild | 2020-04-02 | 1 | N/A | Positive | 91.23 | Positive | Positive |
|  |  |  |  |  | 2020-04-03 | 2 | N/A | Positive | 92.85 | Positive | Positive |
| PT053 | F | 47 | 26.78 | Moderate | 2020-04-02 | 5 | N/A | Positive | 48.28 | Positive | Positive |
| PT054 | M | 38 | 27.69 | Critical | 2020-04-02 | 8 | N/A | Positive | 82.08 | Positive | Positive |
|  |  |  |  |  | 2020-04-04 | 10 | N/A | Positive | 86.24 | Positive | Positive |
|  |  |  |  |  | 2020-04-09 | 15 | N/A | Positive | 92.62 | Positive | Positive |
| PT055 | M | 6 | 20.91 | Mild | 2020-04-04 | 1 | N/A | Positive | 96.29 | Positive | Positive |
| PT056 | F | 41 | 26.28 | Critical | 2020-04-05 | 14 | N/A | Positive | 47.80 | Positive | Positive |
|  |  |  |  |  | 2020-04-09 | 18 | N/A | Positive | 92.48 | Positive | Positive |
| PT057 | F | 33 | 25.32 | Moderate | 2020-04-05 | 7 | N/A | Positive | 86.28 | Positive | Positive |
|  |  |  |  |  | 2020-04-10 | 12 | N/A | Positive | 84.48 | Positive | Positive |
| PT058 | F | 70 | 16.75 | Mild | 2020-04-05 | 5 | N/A | Positive | 91.80 | Positive | Positive |
|  |  |  |  |  | 2020-04-08 | 8 | N/A | Positive | 93.50 | Positive | Positive |
|  |  |  |  |  | 2020-04-09 | 9 | N/A | Positive | 92.26 | Positive | Positive |
| PT059 | F | 33 | 14.49 | Mild | 2020-03-13 | 2 | N/A | Positive | 90.38 | Positive | Positive |
|  |  |  |  |  | 2020-03-24 | 13 | N/A | Positive | 91.79 | Positive | Positive |
| PT060 | F | 50 | 25.82 | Moderate | 2020-03-21 | 6 | 10 | Positive | 85.80 | Positive | Positive |
|  |  |  |  |  | 2020-03-23 | 8 | 12 | Positive | 88.04 | Positive | Positive |
|  |  |  |  |  | 2020-03-27 | 12 | 16 | Positive | 89.80 | Positive | Positive |
| PT061 | F | 29 | 15.2 | Mild | 2020-03-08 | 1 | N/A | Positive | 42.00 | Positive | Positive |
|  |  |  |  |  | 2020-03-20 | 13 | N/A | Positive | 55.22 | Positive | Positive |
|  |  |  |  |  | 2020-03-22 | 15 | N/A | Positive | 89.64 | Positive | Positive |
| PT062 | F | 29 | 18.06 | Mild | 2020-03-11 | 5 | N/A | Positive | 92.95 | Positive | Positive |
|  |  |  |  |  | 2020-03-21 | 15 | N/A | Positive | 92.94 | Positive | Positive |
| PT063 | M | 36 | 15.62 | Mild | 2020-03-12 | 0 | N/A | Positive | 60.61 | Positive | Positive |
|  |  |  |  |  | 2020-03-20 | 8 | N/A | Positive | 61.61 | Positive | Positive |
| PT064 | M | 23 | 12.24 | Mild | 2020-03-15 | 1 | N/A | Positive | 91.12 | Positive | Positive |
|  |  |  |  |  | 2020-03-23 | 9 | N/A | Positive | 92.15 | Positive | Positive |
|  |  |  |  |  | 2020-03-27 | 13 | N/A | Positive | 94.42 | Positive | Positive |
| PT065 | F | 26 | 33.18 | Moderate | 2020-03-16 | 1 | N/A | Positive | 92.73 | Positive | Positive |
|  |  |  |  |  | 2020-03-23 | 8 | N/A | Positive | 95.19 | Positive | Positive |
|  |  |  |  |  | 2020-03-25 | 10 | N/A | Positive | 95.07 | Positive | Positive |
| PT066 | M | 25 | 16.51 | Mild | 2020-03-17 | 2 | 6 | Positive | 51.43 | Positive | Positive |
|  |  |  |  |  | 2020-03-20 | 5 | 9 | Positive | 55.72 | Positive | Positive |
|  |  |  |  |  | 2020-03-23 | 8 | 12 | Positive | 75.92 | Positive | Positive |
|  |  |  |  |  | 2020-03-25 | 10 | 14 | Positive | 77.20 | Positive | Positive |
|  |  |  |  |  | 2020-03-29 | 14 | 18 | Positive | 78.87 | Positive | Positive |
| PT067 | F | 24 | 20.13 | Moderate | 2020-03-18 | 3 | N/A | Positive | 90.89 | Positive | Positive |
|  |  |  |  |  | 2020-03-20 | 5 | N/A | Positive | 91.15 | Positive | Positive |
|  |  |  |  |  | 2020-03-21 | 6 | N/A | Positive | 91.20 | Positive | Positive |
|  |  |  |  |  | 2020-03-27 | 12 | N/A | Positive | 92.24 | Positive | Positive |
|  |  |  |  |  | 2020-03-30 | 15 | N/A | Positive | 93.96 | Positive | Positive |
|  |  |  |  |  | 2020-04-03 | 19 | N/A | Positive | 95.15 | Positive | Positive |
| PT068 | F | 32 | 22.25 | Mild | 2020-03-18 | 1 | 4 | Negative | 11.52 | Positive | Positive |
|  |  |  |  |  | 2020-03-20 | 3 | 7 | Positive | 32.92 | Positive | Positive |
| PT069 | M | 43 | 28.19 | Severe | 2020-03-21 | 2 | N/A | Positive | 87.39 | Positive | Positive |
|  |  |  |  |  | 2020-03-22 | 3 | N/A | Positive | 87.72 | Positive | Positive |
|  |  |  |  |  | 2020-03-24 | 5 | N/A | Positive | 88.91 | Positive | Positive |
|  |  |  |  |  | 2020-03-28 | 9 | N/A | Positive | 89.86 | Positive | Positive |
|  |  |  |  |  | 2020-03-30 | 11 | N/A | Positive | 90.47 | Positive | Positive |
|  |  |  |  |  | 2020-04-03 | 15 | N/A | Positive | 91.63 | Positive | Positive |
|  |  |  |  |  | 2020-04-05 | 17 | N/A | Positive | 92.61 | Positive | Positive |
| PT070 | M | 48 | 17.09 | Critical | 2020-03-23 | 3 | N/A | Positive | 66.63 | Positive | Positive |
|  |  |  |  |  | 2020-03-24 | 4 | N/A | Positive | 69.01 | Positive | Positive |
|  |  |  |  |  | 2020-03-28 | 8 | N/A | Positive | 79.36 | Positive | Positive |
|  |  |  |  |  | 2020-03-30 | 10 | N/A | Positive | 88.52 | Positive | Positive |
|  |  |  |  |  | 2020-03-31 | 11 | N/A | Positive | 87.14 | Positive | Positive |
|  |  |  |  |  | 2020-04-03 | 14 | N/A | Positive | 93.48 | Positive | Positive |
|  |  |  |  |  | 2020-04-16 | 27 | N/A | Positive | 82.53 | Positive | Positive |
|  |  |  |  |  | 2020-04-19 | 30 | N/A | Positive | 97.12 | Positive | Positive |
| PT071 | M | 54 | 23.56 | Moderate | 2020-03-27 | 9 | 16 | Positive | 88.12 | Positive | Positive |
|  |  |  |  |  | 2020-03-29 | 11 | 18 | Positive | 90.18 | Positive | Positive |
|  |  |  |  |  | 2020-04-02 | 15 | 22 | Positive | 90.89 | Positive | Positive |
| PT072 | F | 24 | 23.94 | Severe | 2020-03-29 | 2 | N/A | Positive | 89.74 | Positive | Positive |
|  |  |  |  |  | 2020-04-02 | 6 | N/A | Positive | 90.89 | Positive | Positive |
|  |  |  |  |  | 2020-04-03 | 7 | N/A | Positive | 91.70 | Positive | Positive |
| PT073 | M | 57 | 22.29 | Critical | 2020-04-04 | 4 | N/A | Positive | 91.70 | Positive | Positive |
|  |  |  |  |  | 2020-04-07 | 7 | N/A | Positive | 95.18 | Positive | Positive |
| PT074 | F | N/A | N/A | Mild | 2020-04-01 | 0 | N/A | Positive | 62.07 | Positive | Positive |
| PT075 | M | 46 | 29.98 | Severe | 2020-04-02 | 1 | N/A | Positive | 78.60 | Positive | Positive |
|  |  |  |  |  | 2020-04-04 | 3 | N/A | Positive | 85.48 | Positive | Positive |
|  |  |  |  |  | 2020-04-06 | 5 | N/A | Positive | 90.55 | Positive | Positive |
|  |  |  |  |  | 2020-04-11 | 10 | N/A | Positive | 92.40 | Positive | Positive |
|  |  |  |  |  | 2020-04-13 | 12 | N/A | Positive | 94.76 | Positive | Positive |
| PT076 | M | 34 | 14.24 | Mild | 2020-04-04 | 1 | N/A | Positive | 73.84 | Positive | Positive |
|  |  |  |  |  | 2020-04-06 | 3 | N/A | Positive | 90.18 | Positive | Positive |
| PT077 | F | 50 | 19.68 | Moderate | 2020-04-05 | 2 | N/A | Positive | 40.99 | Positive | Positive |
|  |  |  |  |  | 2020-04-07 | 4 | N/A | Positive | 43.11 | Positive | Positive |
|  |  |  |  |  | 2020-04-09 | 6 | N/A | Positive | 46.41 | Positive | Positive |
| PT078 | M | 58 | 29.18 | Severe | 2020-04-05 | 8 | N/A | Positive | 53.39 | Positive | Positive |
|  |  |  |  |  | 2020-04-10 | 13 | N/A | Positive | 86.18 | Positive | Positive |
|  |  |  |  |  | 2020-04-15 | 18 | N/A | Positive | 95.45 | Positive | Positive |
| PT079 | M | 49 | 24.9 | Severe | 2020-04-09 | 7 | 14 | Positive | 96.21 | Positive | Positive |
|  |  |  |  |  | 2020-04-12 | 10 | 17 | Positive | 81.00 | Positive | Positive |
|  |  |  |  |  | 2020-04-17 | 15 | 22 | Positive | 95.66 | Positive | Positive |
|  |  |  |  |  | 2020-04-23 | 21 | 28 | Positive | 96.21 | Positive | Positive |
| PT080 | M | N/A | N/A | Mild | 2020-04-10 | 16 | N/A | Positive | 94.61 | Positive | Positive |
|  |  |  |  |  | 2020-04-15 | 21 | N/A | Positive | 96.91 | Positive | Positive |
|  |  |  |  |  | 2020-04-20 | 26 | N/A | Positive | 96.65 | Positive | Positive |
| PT081 | M | 43 | 30.7 | Severe | 2020-04-10 | 3 | N/A | Positive | 37.46 | Positive | Positive |
|  |  |  |  |  | 2020-04-15 | 8 | N/A | Positive | 96.53 | Positive | Positive |
| PT082 | F | 74 | 15.2 | Severe | 2020-04-11 | 1 | N/A | Positive | 42.39 | Positive | Positive |
|  |  |  |  |  | 2020-04-14 | 4 | N/A | Positive | 41.94 | Positive | Positive |
|  |  |  |  |  | 2020-04-19 | 9 | N/A | Positive | 53.60 | Positive | Positive |
|  |  |  |  |  | 2020-04-24 | 14 | N/A | Positive | 96.91 | Positive | Positive |
| PT083 | F | 49 | 24.93 | Moderate | 2020-04-12 | 11 | N/A | Positive | 49.11 | Positive | Positive |
|  |  |  |  |  | 2020-04-15 | 14 | N/A | Positive | 88.65 | Positive | Positive |
| PT084 | F | 38 | 31.1 | Moderate | 2020-04-13 | 1 | N/A | Positive | 59.04 | Positive | Positive |
|  |  |  |  |  | 2020-04-23 | 11 | N/A | Positive | 73.23 | Positive | Positive |
| PT085 | F | N/A | N/A | Mild | 2020-04-19 | 2 | N/A | Positive | 71.40 | Positive | Positive |
| PT086 | F | 34 | 18.22 | Critical | 2020-04-13 | 9 | N/A | Positive | 92.86 | Positive | Positive |
|  |  |  |  |  | 2020-04-17 | 13 | N/A | Positive | 93.23 | Positive | Positive |
| PT087 | F | 11 | 24.32 | Mild | 2020-04-14 | 1 | N/A | Positive | 23.62 | Positive | Positive |
| PT088 | F | 38 | 28.03 | Mild | 2020-04-14 | 5 | N/A | Positive | 80.77 | Positive | Positive |
|  |  |  |  |  | 2020-04-15 | 6 | N/A | Positive | 83.29 | Positive | Positive |
| PT089 | M | 38 | 27.69 | Critical | 2020-04-14 | 8 | N/A | Positive | 96.68 | Positive | Positive |
| PT090 | F | 35 | N/A | Severe | 2020-04-15 | 1 | N/A | Positive | 79.76 | Positive | Positive |
|  |  |  |  |  | 2020-04-21 | 7 | N/A | Positive | 91.02 | Positive | Positive |
|  |  |  |  |  | 2020-04-23 | 9 | N/A | Positive | 93.46 | Positive | Positive |
| PT091 | F | 24 | 21.93 | Mild | 2020-03-16 | 1 | N/A | Positive | 44.28 | Positive | Positive |
| PT092 | F | N/A | N/A | Mild | 2020-04-16 | 5 | N/A | Positive | 41.20 | Positive | Positive |
|  |  |  |  |  | 2020-04-19 | 8 | N/A | Positive | 76.92 | Positive | Positive |
|  |  |  |  |  | 2020-04-24 | 13 | N/A | Positive | 92.67 | Positive | Positive |
| PT093 | F | N/A | N/A | Mild | 2020-04-16 | 4 | N/A | Positive | 34.76 | Positive | Positive |
|  |  |  |  |  | 2020-04-18 | 6 | N/A | Positive | 66.70 | Positive | Positive |
|  |  |  |  |  | 2020-04-19 | 7 | N/A | Positive | 85.51 | Positive | Positive |
|  |  |  |  |  | 2020-04-20 | 8 | N/A | Positive | 91.02 | Positive | Positive |
|  |  |  |  |  | 2020-04-24 | 12 | N/A | Positive | 96.34 | Positive | Positive |
|  |  |  |  |  | 2020-04-26 | 14 | N/A | Positive | 94.31 | Positive | Positive |
| PT094 | F | 49 | 26.44 | Severe | 2020-04-18 | 1 | N/A | Positive | 46.16 | Positive | Positive |
|  |  |  |  |  | 2020-04-20 | 3 | N/A | Positive | 56.47 | Positive | Positive |
|  |  |  |  |  | 2020-04-25 | 8 | N/A | Positive | 62.81 | Positive | Positive |
| PT095 | M | 24 | 16.5 | Mild | 2020-04-19 | 2 | N/A | Positive | 46.24 | Positive | Positive |
|  |  |  |  |  | 2020-04-22 | 5 | N/A | Positive | 65.38 | Positive | Positive |
| PT096 | M | 69 | 25.14 | Moderate | 2020-04-03 | 5 | N/A | Positive | 66.01 | Positive | Positive |
|  |  |  |  |  | 2020-04-04 | 6 | N/A | Positive | 70.58 | Positive | Positive |
|  |  |  |  |  | 2020-04-05 | 7 | N/A | Positive | 77.58 | Positive | Positive |
|  |  |  |  |  | 2020-04-07 | 9 | N/A | Positive | 79.92 | Positive | Positive |
| PT097 | F |  | 15.91 | Mild | N/A | N/A | N/A | Positive | 90.61 | Positive | Positive |
