# Supplementary material for: Antibodies response in symptomatic and asymptomatic SARS-CoV-2 infected persons in Thailand
Source: PLoS One. 2025 Feb 11;20(2):e0308850. doi: 10.1371/journal.pone.0308850 (PMC11813072; doi:10.1371/journal.pone.0308850)
Supplement: S2 Table — (DOCX) [file pone.0308850.s007.docx]

S7 Table. This is the Table2 Shows the results of samples from State Quarantine, from persons travelling back from Sudan in the latter half of 2020 tested using rRT-PCR, ELISA IgG, IgM and sVNT.

| Patient ID | Interpretation | Day  (0, 3, 5, 7, 9, 11) | rRT-PCR Result from Test Kit 1 | | rRT-PCR Result from Test Kit 2 | | | ELISA Results | | | | |
| --- | --- | --- | --- | --- | --- | --- | --- | --- | --- | --- | --- | --- |
|  |  |  | RdRP | N gene | E gene | RdRP | N gene | Day | Neutralization Ab (sVNT) | % Inhibition | IgM | IgG |
| SD01 | Not detected | - | - | - | - | - | - | 0 | Negative | 12.81 | Negative | Negative |
|  |  |  |  |  |  |  |  | 14 | Negative | 0.03 | Negative | Negative |
| SD02 | Not detected | - | - | - | - | - | - | 0 | **Positive** | 53.36 | Negative | **Positive** |
|  |  |  |  |  |  |  |  | 14 | **Positive** | 33.68 | Negative | **Positive** |
| SD03 | Not detected | - | - | - | - | - | - | 0 | Negative | 19.13 | Negative | Negative |
|  |  |  |  |  |  |  |  | 14 | Negative | 5.33 | Negative | Negative |
| SD04 | Not detected | - | - | - | - | - | - | 0 | Negative | 13.02 | Negative | Negative |
|  |  |  |  |  |  |  |  | 14 | Negative | 8.34 | Negative | Negative |
| SD05 | Not detected | - | - | - | - | - | - | 0 | Negative | 10.14 | Negative | Negative |
|  |  |  |  |  |  |  |  | 14 | Negative | 3.73 | Negative | Negative |
| SD06 | Not detected | - | - | - | - | - | - | 0 | Negative | 9.16 | Negative | Negative |
|  |  |  |  |  |  |  |  | 14 | Negative | 5.85 | Negative | Negative |
| SD07 | Not detected | - | - | - | - | - | - | 0 | **Positive** | 47.41 | Negative | **Positive** |
|  |  |  |  |  |  |  |  | 14 | **Positive** | 34.09 | Negative | **Positive** |
| SD08 | Detected | Day 3 | 37.27 | 35.41 | - | - | - | 0 | **Positive** | 47.06 | Negative | **Positive** |
|  |  |  |  |  |  |  |  | 14 | N/S | N/S | N/S | N/S |
| SD09 | Detected | Day 5 | - | - | 31.95 | 33.99 | 34.14 | 0 | **Positive** | 76.18 | Negative | **Positive** |
|  |  |  |  |  |  |  |  | 14 | N/S | N/S | N/S | N/S |
| SD10 | Not detected | - | - | - | - | - | - | 0 | Negative | 12.36 | Negative | Negative |
|  |  |  |  |  |  |  |  | 14 | Negative | 3.73 | Negative | Negative |
| SD11 | Not detected | - | - | - | - | - | - | 0 | Negative | 18.36 | Negative | Negative |
|  |  |  |  |  |  |  |  | 14 | Negative | 5.77 | Negative | Negative |
| SD12 | Not detected | - | - | - | - | - | - | 0 | **Positive** | 89.35 | Negative | **Positive** |
|  |  |  |  |  |  |  |  | 14 | **Positive** | 72.73 | **Positive** | **Positive** |
| SD13 | Not detected | - | - | - | - | - | - | 0 | Negative | 15.56 | Negative | Negative |
|  |  |  |  |  |  |  |  | 14 | Negative | 9.80 | Negative | Negative |
| SD14 | Not detected | - | - | - | - | - | - | 0 | **Positive** | 76.63 | Negative | **Positive** |
|  |  |  |  |  |  |  |  | 14 | **Positive** | 60.06 | Negative | **Positive** |
| SD15 | Not detected | - | - | - | - | - | - | 0 | **Positive** | 87.57 | Negative | **Positive** |
|  |  |  |  |  |  |  |  | 14 | **Positive** | 57.79 | Negative | **Positive** |
| SD16 | Not detected | - | - | - | - | - | - | 0 | Negative | 12.65 | Negative | Negative |
|  |  |  |  |  |  |  |  | 14 | Negative | 1.20 | Negative | Negative |
| SD17 | Not detected | - | - | - | - | - | - | 0 | Negative | 10.96 | Negative | Negative |
|  |  |  |  |  |  |  |  | 14 | Negative | 0.63 | Negative | Negative |
| SD18 | Not detected | - | - | - | - | - | - | 0 | Negative | 8.73 | Negative | Negative |
|  |  |  |  |  |  |  |  | 14 | Negative | 2.54 | Negative | Negative |
| SD19 | Not detected | - | - | - | - | - | - | 0 | **Positive** | 84.24 | Negative | **Positive** |
|  |  |  |  |  |  |  |  | 14 | **Positive** | 59.15 | **Positive** | **Positive** |
|  |  |  |  |  |  |  |  |  |  |  |  |  |
| SD20 | Not detected | - | - | - | - | - | - | 0 | Negative | 11.17 | Negative | Negative |
|  |  |  |  |  |  |  |  | 14 | Negative | 4.10 | Negative | Negative |
| SD21 | Not detected | - | - | - | - | - | - | 0 | Negative | 17.60 | Negative | Negative |
|  |  |  |  |  |  |  |  | 14 | Negative | 3.31 | Negative | Negative |
| SD22 | Not detected | - | - | - | - | - | - | 0 | Negative | 13.02 | Negative | Negative |
|  |  |  |  |  |  |  |  | 14 | Negative | 0.56 | Negative | Negative |
| SD23 | Not detected | - | - | - | - | - | - | 0 | Negative | 9.52 | Negative | Negative |
|  |  |  |  |  |  |  |  | 14 | Negative | 11.41 | Negative | Negative |
| SD24 | Not detected | - | - | - | - | - | - | 0 | **Positive** | 61.00 | Negative | **Positive** |
|  |  |  |  |  |  |  |  | 14 | **Positive** | 35.44 | **Positive** | **Positive** |
| SD25 | Not detected | - | - | - | - | - | - | 0 | Negative | 11.36 | Negative | Negative |
|  |  |  |  |  |  |  |  | 14 | Negative | 11.34 | Negative | Negative |
| SD26 | Not detected | - | - | - | - | - | - | 0 | Negative | 13.39 | Negative | Negative |
|  |  |  |  |  |  |  |  | 14 | Negative | 7.62 | Negative | Negative |
| SD27 | Detected | Day 3 | 39.44 | 35.56 | - | - | 37.81 | 0 | **Positive** | 58.28 | Negative | **Positive** |
|  |  |  |  |  |  |  |  | 14 | N/S | N/S | N/S | N/S |
| SD28 | Not detected | - | - | - | - | - | - | 0 | Negative | 17.28 | Negative | Negative |
|  |  |  |  |  |  |  |  | 14 | Negative | 2.43 | Negative | Negative |
| SD29 | Not detected | - | - | - | - | - | - | 0 | Negative | 9.02 | Negative | Negative |
|  |  |  |  |  |  |  |  | 14 | Negative | 11.28 | Negative | Negative |
|  |  |  |  |  |  |  |  |  |  |  |  |  |
| SD30 | Not detected | - | - | - | - | - | - | 0 | **Positive** | 71.47 | Negative | **Positive** |
|  |  |  |  |  |  |  |  | 14 | Negative | 1.47 | Negative | Negative |
| SD31 | Not detected | - | - | - | - | - | - | 0 | Negative | 12.41 | Negative | Negative |
|  |  |  |  |  |  |  |  | 14 | Negative | 11.18 | Negative | Negative |
| SD32 | Detected | Day 0 | 34.27 | 34.26 | 33.78 | 35.83 | 35.65 | 0 | Negative | 10.98 | Negative | Negative |
|  |  |  |  |  |  |  |  | 14 | N/S | N/S | Negative | **Positive** |
| SD33 | Not detected | - | - | - | - | - | - | 0 | **Positive** | 56.31 | Negative | **Positive** |
|  |  |  |  |  |  |  |  | 14 | **Positive** | 56.41 | Negative | **Positive** |
| SD34 | Not detected | - | - | - | - | - | 39.25 | 0 | **Positive** | 82.12 | Negative | **Positive** |
|  |  |  |  |  |  |  |  | 14 | **Positive** | 60.66 | Negative | **Positive** |
| SD35 | Not detected | - | - | - | - | - |  | 0 | **Positive** | 84.08 | Negative | **Positive** |
|  |  |  |  |  |  |  |  | 14 | Negative | 2.82 | Negative | Negative |
| SD36 | Not detected | - | - | - | - | - |  | 0 | Negative | 11.63 | Negative | Negative |
|  |  |  |  |  |  |  |  | 14 | Negative | 5.72 | Negative | Negative |
| SD37 | Not detected | - | - | - | - | - |  | 0 | Negative | 12.70 | Negative | Negative |
|  |  |  |  |  |  |  |  | 14 | Negative | 3.74 | Negative | Negative |
| SD38 | Not detected | - | - | - | - | - | - | 0 | Negative | 9.82 | Negative | Negative |
|  |  |  |  |  |  |  |  | 14 | **Positive** | 40.46 | Negative | Negative |
| SD39 | Detected | Day 9 | - | - | 37.09 | 38.23 | 37.71 | 0 | **Positive** | 60.75 | Negative | **Positive** |
|  |  |  |  |  |  |  |  | 14 | **Positive** | 68.70 | Negative | **Positive** |
|  |  |  |  |  |  |  |  |  |  |  |  |  |
| SD40 | Not detected | - | - | - | - | - | - | 0 | **Positive** | 91.11 | Negative | **Positive** |
|  |  |  |  |  |  |  |  | 14 | Negative | 3.89 | Negative | Negative |
| SD41 | Not detected | - | - | - | - | - |  | 0 | Negative | 8.48 | Negative | Negative |
|  |  |  |  |  |  |  |  | 14 | Negative | 9.24 | Negative | Negative |
| SD42 | Invalid internal control | - | - | - | - | - | 36.79 | 0 | Negative | 8.10 | Negative | Negative |
|  |  |  |  |  |  |  |  | 14 | N/S | N/S | Negative | Negative |
| SD43 | Not detected | - | - | - | - | - |  | 0 | Negative | 15.14 | Negative | Negative |
|  |  |  |  |  |  |  |  | 14 | Negative | 4.99 | Negative | Negative |
| SD44 | Not detected | - | - | - | - | - |  | 0 | Negative | 10.15 | Negative | Negative |
|  |  |  |  |  |  |  |  | 14 | Negative | 1.10 | Negative | Negative |
| SD45 | Detected | Day 3 | 34.87 | 32.76 | - | - | - | 0 | **Positive** | 49.96 | Negative | **Positive** |
|  |  |  |  |  |  |  |  | 14 | N/S | N/S | N/S | N/S |
| SD46 | Detected | Day 0 | 32.09 | 32.38 | 29.01 | 31.16 | 32.05 | 0 | Negative | 7.56 | Negative | Negative |
|  |  |  |  |  |  |  |  | 14 | N/S | N/S | Negative | **Positive** |
| SD47 | Not detected | - | - | - | - | - | - | 0 | **Positive** | 92.99 | Negative | **Positive** |
|  |  |  |  |  |  |  |  | 14 | **Positive** | 73.47 | Negative | **Positive** |
| SD48 | Not detected | - | - | - | - | - | - | 0 | Negative | 2.44 | Negative | Negative |
|  |  |  |  |  |  |  |  | 14 | Negative | 9.21 | Negative | Negative |
| SD49 | Detected | Day 3 | 34.43 | 32.83 | - | - | - | 0 | **Positive** | 64.31 | Negative | **Positive** |
|  |  |  |  |  |  |  |  | 14 | N/S | N/S | N/S | N/S |
|  |  |  |  |  |  |  |  |  |  |  |  |  |
| SD50 | Not detected | - | - | - | - | - | - | 0 | Negative | 10.63 | Negative | Negative |
|  |  |  |  |  |  |  |  | 14 | Negative | 12.58 | Negative | Negative |
| SD51 | Not detected | - | - | - | - | - | - | 0 | Negative | 10.57 | Negative | Negative |
|  |  |  |  |  |  |  |  | 14 | Negative | 1.48 | Negative | Negative |
| SD52 | Not detected | - | - | - | - | - | - | 0 | Negative | 10.72 | Negative | Negative |
|  |  |  |  |  |  |  |  | 14 | Negative | 3.68 | Negative | Negative |
| SD53 | Not detected | - | - | - | - | - | - | 0 | Negative | 11.94 | Negative | Negative |
|  |  |  |  |  |  |  |  | 14 | Negative | 6.94 | Negative | Negative |
| SD54 | Detected | Day 5 | - | - | - | 38.6 | 37.07 | 0 | Negative | 16.72 | Negative | Negative |
|  |  |  |  |  |  |  |  | 14 | N/S | N/S | **Positive** | **Positive** |
| SD55 | Detected | Day 5 | - | - | 34.78 | 35.33 | 35.36 | 0 | **Positive** | 65.75 | Negative | **Positive** |
|  |  |  |  |  |  |  |  | 14 | N/S | N/S | N/S | N/S |
| SD56 | Detected | Day 0 | 34.51 | 31.21 | 30.2 | 32.94 | 31.52 | 0 | Negative | 3.13 | Negative | Negative |
|  |  |  |  |  |  |  |  | 14 | N/S | N/S | **Positive** | **Positive** |
| SD57 | Not detected | - | - | - | - | - | - | 0 | **Positive** | 52.76 | Negative | **Positive** |
|  |  |  |  |  |  |  |  | 14 | **Positive** | 44.10 | Negative | **Positive** |
| SD58 | Not detected | - | - | - | - | - | - | 0 | **Positive** | 68.54 | Negative | **Positive** |
|  |  |  |  |  |  |  |  | 14 | **Positive** | 37.94 | Negative | **Positive** |
| SD59 | Not detected | - | - | - | - | - | - | 0 | Negative | 10.43 | Negative | Negative |
|  |  |  |  |  |  |  |  | 14 | Negative | 6.65 | Negative | Negative |
|  |  |  |  |  |  |  |  |  |  |  |  |  |
| SD60 | Not detected | - | - | - | - | - | - | 0 | Positive | 53.40 | Negative | Positive |
|  |  |  |  |  |  |  |  | 14 | Negative | 6.31 | Negative | Negative |
| SD61 | Not detected | - | - | - | - | - | - | 0 | Positive | 56.39 | Negative | Positive |
|  |  |  |  |  |  |  |  | 14 | Positive | 35.60 | Negative | Positive |
| SD62 | Not detected |  | - | - | - | - | - | 0 | Negative | 9.54 | Negative | Negative |
|  |  |  |  |  |  |  |  | 14 | Negative | 5.61 | Negative | Negative |
| SD63 | Not detected | - | - | - | - | - | - | 0 | Negative | 19.88 | Negative | Negative |
|  |  |  |  |  |  |  |  | 14 | Negative | 12.57 | Negative | Negative |
| SD64 | Detected | Day 0 | 35.39 | 33.24 | - | - | - | 0 | Positive | 50.10 | Negative | Positive |
|  |  |  |  |  |  |  |  | 14 | N/S | N/S | N/S | N/S |
| SD65 | Not detected | - | - | - | - | - | - | 0 | Negative | 14.64 | Negative | Negative |
|  |  |  |  |  |  |  |  | 14 | Negative | 7.95 | Negative | Negative |
| SD66 | Not detected | - | - | - | - | - | - | 0 | Positive | 66.84 | Negative | Positive |
|  |  |  |  |  |  |  |  | 14 | Positive | 38.95 | Negative | Positive |
| SD67 | Not detected | - | - | - | - | - | - | 0 | Negative | 12.69 | Negative | Negative |
|  |  |  |  |  |  |  |  | 14 | Negative | 4.11 | Negative | Negative |
| SD68 | Detected | Day 0 | 33.36 | 33.04 | 29.33 | 31.41 | 32.02 | 0 | Positive | 88.71 | Negative | Positive |
|  |  |  |  |  |  |  |  | 14 | N/S | N/S | N/S | N/S |
| SD69 | Not detected | - | - | - | - | - | - | 0 | Negative | 8.04 | Negative | Negative |
|  |  |  |  |  |  |  |  | 14 | Negative | 11.06 | Negative | Negative |
|  |  |  |  |  |  |  |  |  |  |  |  |  |
| SD70 | Not detected | - | - | - | - | - | - | 0 | Negative | 7.79 | Negative | Negative |
|  |  |  |  |  |  |  |  | 14 | Negative | 2.72 | Negative | Negative |
| SD71 | Not detected | - | - | - | - | - | - | 0 | Negative | 12.66 | Negative | Negative |
|  |  |  |  |  |  |  |  | 14 | Negative | 0.07 | Negative | Negative |
| SD72 | Not detected | - | - | - | - | - | - | 0 | Negative | 9.17 | Negative | Negative |
|  |  |  |  |  |  |  |  | 14 | Negative | 4.12 | Negative | Negative |
| SD73 | Detected | Day 3 | 39.13 | 36.02 | - | - | - | 0 | Positive | 74.93 | Negative | Positive |
|  |  |  |  |  |  |  |  | 14 | N/S | N/S | N/S | N/S |
| SD74 | Not detected | - | - | - | - | - | - | 0 | Negative | 8.32 | Negative | Negative |
|  |  |  |  |  |  |  |  | 14 | Negative | 6.32 | Negative | Negative |
| SD75 | Detected | Day 3 | 38.37 | 34.7 | - | - | - | 0 | Positive | 46.07 | Negative | Positive |
|  |  |  |  |  |  |  |  | 14 | N/S | N/S | N/S | N/S |
| SD76 | Not detected | - | - | - | - | - | - | 0 | Negative | 13.41 | Negative | Negative |
|  |  |  |  |  |  |  |  | 14 | Negative | 7.75 | Negative | Negative |
| SD77 | Not detected | - | - | - | - | - | - | 0 | Negative | 10.43 | Negative | Negative |
|  |  |  |  |  |  |  |  | 14 | Negative | 0.29 | Negative | Negative |

N/S: Specimen was not sufficient for sVNT and other assays as other laboratory tests needed to be conducted
