# Supplementary material for: Antibodies response in symptomatic and asymptomatic SARS-CoV-2 infected persons in Thailand
Source: PLoS One. 2025 Feb 11;20(2):e0308850. doi: 10.1371/journal.pone.0308850 (PMC11813072; doi:10.1371/journal.pone.0308850)
Supplement: S3 Table — (DOCX) [file pone.0308850.s008.docx]

S8 Table. This is the Table3 Shows the results of Analyses of 97 PCR-positive COVID-19 patients against % inhibition of sVNT for the different disease severity categories

| Dependent Variable: % Inhibition | | | | | | |
| --- | --- | --- | --- | --- | --- | --- |
| LSD |  |  |  |  |  |  |
| (I) Disease Severity | (J) Disease Severity | Mean Difference (I-J) | Std. Error | Sig. | 95% Confidence Interval | |
|  |  |  |  |  | Lower Bound | Upper Bound |
| Critical | Mild | 10.45 | 4.29 | **0.02*** | 2.00 | 18.91 |
|  | Moderate | 4.39 | 4.56 | 0.34 | -4.59 | 13.37 |
|  | Severe | 5.28 | 4.73 | 0.27 | -4.04 | 14.60 |
| Mild | Critical | -10.45* | 4.29 | **0.02*** | -18.91 | -2.00 |
|  | Moderate | -6.07 | 3.33 | 0.07 | -12.63 | 0.49 |
|  | Severe | -5.18 | 3.57 | 0.15 | -12.20 | 1.85 |
| Moderate | Critical | -4.39 | 4.56 | 0.34 | -13.37 | 4.59 |
|  | Mild | 6.07 | 3.33 | 0.07 | -0.49 | 12.63 |
|  | Severe | 0.89 | 3.88 | 0.82 | -6.75 | 8.54 |
| Severe | Critical | -5.28 | 4.73 | 0.27 | -14.60 | 4.04 |
|  | Mild | 5.18 | 3.57 | 0.15 | -1.85 | 12.20 |
|  | Moderate | -0.89 | 3.88 | 0.82 | -8.54 | 6.75 |
| * The mean difference is significant at the 0.05 level. | | | | | | |
