# Supplementary material for: Antibodies response in symptomatic and asymptomatic SARS-CoV-2 infected persons in Thailand
Source: PLoS One. 2025 Feb 11;20(2):e0308850. doi: 10.1371/journal.pone.0308850 (PMC11813072; doi:10.1371/journal.pone.0308850)
Supplement: Attachment — Submitted filename: Response to Reviewers latter_edit.docx [file pone.0308850.s009.docx]

Edit submission Cover Letter

## Answers to editor PONE-D-23-25209 - [EMID:f91d004e9f54cbb2]

Title: Antibodies response in symptomatic and asymptomatic SARS-CoV-2 infected persons in Thailand

Dear Editor,

We sincerely thank you for reviewing our manuscript and for the excellent suggestions we received We have made a concerted effort to improve it and agree with all the changes that were made.

Regarding the question of whether this manuscript is a resubmission of PONE-D-22-18026R1, we apologize for any confusion. We were unable to access the link [http://journals.plos.org/plosone/s/file?id=dc11/PLOS] because the system reported an error and the steps taken to address the problem. As a result, we have attached a data availability statement for the manuscript, which is part of the supporting information.

In this revised version, the sentences are clearer, and the sequence of events and explanations is more organized.

Best regards,

Chanida Ruchisrisarod

Corresponding author
