# Supplementary material for: Antibodies response in symptomatic and asymptomatic SARS-CoV-2 infected persons in Thailand
Source: PLoS One. 2025 Feb 11;20(2):e0308850. doi: 10.1371/journal.pone.0308850 (PMC11813072; doi:10.1371/journal.pone.0308850)
Supplement: Attachment — Submitted filename: Response to Reviewers latter_reversion.docx [file pone.0308850.s010.docx]

Edit submission Cover Letter

## Answers to editor PONE-D-23-25209 - [EMID:f91d004e9f54cbb2]

Title: Antibodies response in symptomatic and asymptomatic SARS-CoV-2 infected persons in Thailand

Dear Editor,

We sincerely thank you for reviewing our manuscript and for the excellent suggestions we received We have made a concerted effort to improve it and agree with all the changes that were made.

We edit accordingly by reviewers' comments and include statistical analysis information to make it easier to understand. Summarize Table 2 to make it easier to understand and reduce excessive use of data. In this revised version, the sentences are clearer, and the sequence of events and explanations is more organized.

Best regards,

Chanida Ruchisrisarod

Corresponding author
