# Supplementary material for: Antibodies response in symptomatic and asymptomatic SARS-CoV-2 infected persons in Thailand
Source: PLoS One. 2025 Feb 11;20(2):e0308850. doi: 10.1371/journal.pone.0308850 (PMC11813072; doi:10.1371/journal.pone.0308850)
Supplement: Attachment — Submitted filename: Response to Reviewers latter_reversion2.docx [file pone.0308850.s011.docx]

Edit submission Cover Letter

## Answers to editor PONE-D-23-25209 - [EMID:f91d004e9f54cbb2]

Title: Antibodies response in symptomatic and asymptomatic SARS-CoV-2 infected persons in Thailand

Dear Editor,

We sincerely appreciate your thorough review of our manuscript and the valuable suggestions provided. We have diligently worked to enhance the manuscript and have incorporated all the recommended changes. Additionally, we have included statistical analysis information to enhance clarity. To streamline the presentation, we have summarized Table 1 and Table 2 for easier understanding and reduced the excessive use of data.

We have also added a demographic table to facilitate comparison between each group. Furthermore, we have provided an explanation of the diagnosis of asymptomatic patients. In this revised version, the sentences are clearer, and the sequence of events and explanations is more organized.

Best regards,

Chanida Ruchisrisarod

Corresponding author
