# Supplementary material for: Antibodies response in symptomatic and asymptomatic SARS-CoV-2 infected persons in Thailand
Source: PLoS One. 2025 Feb 11;20(2):e0308850. doi: 10.1371/journal.pone.0308850 (PMC11813072; doi:10.1371/journal.pone.0308850)
Supplement: Attachment — Submitted filename: SARS.docx [file pone.0308850.s012.docx]

**Poor English in many situations**

**SARS-CoV-2 antibody detection:**

ELISA in-house kit testing for IgG, IgM and sVNT were developed:

Validation of in-house kit should be done before use in real tests. In text this not sated.

Sources of materials are not indicated.

Preparation of ELISA plate and running of the test are merged. Preparation and validation of the in-house prepared kit should be done first and then being used to test sera.

Please verify in-house kit and then us in test separately.

Also, IgM detection is badly written.

“Fifty μl of TMB substrate was added to each well and incubated in a dark room at room temperature. Wells were washed five times with PBST 300μl per well, after which the reaction was stopped using sulphuric acid.” This absolutely cannot be done in ELISA technique.

Validation of the in-house kits is indicated at the end of the method section.

As the ELISA technique seems to be run with a wrong way, it is not wise to continue reviewing the manuscript. ELISA is the core method in this work which measures antibodies (IgG & IgM) which are the principles of the manuscript title
